# What's a SNP between friends: The lineage of *Clostridioides difficile* R20291 can effect research outcomes

**DOI:** 10.1016/j.anaerobe.2021.102422

**Published:** 2021-10

**Authors:** Jorge Monteford, Terry W. Bilverstone, Patrick Ingle, Sheryl Philip, Sarah A. Kuehne, Nigel P. Minton

**Affiliations:** a Clostridia Research Group, BBSRC/EPSRC Synthetic Biology Research Centre (SBRC), School of Life Sciences, Biodiscovery Institute, The University of Nottingham, Nottingham, NG7 2RD, UK; b NIHR Nottingham Biomedical Research Centre, Nottingham University Hospitals NHS Trust and the University of Nottingham, Nottingham, NG7 2RD, UK; c Biocatalysts Limited, Unit 1, Cefn Coed, Parc Nantgarw, Cardiff, CF15 7QQ, UK; d Oral Microbiology Group, School of Dentistry and Institute of Microbiology and Infection, College of Medical and Dental Sciences, The University of Birmingham, Birmingham, B5 7EG, UK

**Keywords:** *Clostridioides difficile* R20291, Motility, Biofilm, Toxin production, Conjugation, Genomic variation

## Abstract

*Clostridioides difficile* R20291 is the most studied PCR-Ribotype 027 isolate. The two predominant lineages of this hypervirulent strain, however, exhibit substantive phenotypic differences and possess genomes that differ by a small number of nucleotide changes. It is important that the source of R20291 is taken into account in research outcomes.

## Highlights

• Two phenotypically distinct lineages of *C. difficile* R20291 are in circulation.
• Affected are gene transfer, motility, sporulation, biofilm and toxin production.
• The differences are due to a small number of single nucleotide genomic changes.
• These findings underline the importance of appropriately maintaining stock cultures.
• The source of R20291 should be stated in any investigation.

*Clostridioides difficile* (formerly *Clostridium difficile* [1]) is the leading cause of hospital-associated diarrhoea in the developed world. Its prevalence in recent years has been attributed to the emergence of hypervirulent strains, and in particular those belonging to BI/NAP1/PCR ribotype 027 (RT 027) which elaborate high titres of Toxin A/B, produce binary toxin and exhibit an increased propensity to form spores [2]. The first RT 027 strain to have its genome sequenced was strain R20291 [3] responsible for a major outbreak in 2006 at Stoke Mandeville Hospital, UK. Consequently, R20291 has become one of the most studied laboratory strains of *C. difficile.*

Full exploitation of clostridial genome sequence data has relied on the application of forward and reverse genetics tools [4], most notably ClosTron technology based on intron re-targeting [5]. Initial attempts to generate mutants in R20291, however, found that the effective transfer of the ClosTron plasmid was dependent on the R20291 stock used. Transfer was reproducibly possible using CRG0825, a stock of R20291 obtained by Nottingham's Clostridia Research Group (CRG) from the UK Anaerobe reference unit (ARU), Cardiff, UK. In comparison, transfer to a stock of R20291 (CRG2021) originating from the Brendon Wren laboratory at The London School of Hygiene and Tropical Medicine (LSHTM), was extremely ineffective. Consequently, the CRG0825 was taken forward in reverse genetic studies using the ClosTron and as the basis for the development of allelic-exchange (AE) technology based on *pyrE* alleles [6]. As a result, CRG0825 and its Δ*pyrE* derivative have been widely distributed to research laboratories wishing to study R20291.

The inefficient nature of CRG2021 as a conjugative recipient is not confined to the ClosTron plasmid but affects a range of different vectors which are transferred to CRG0825 at rates that are an order of magnitude higher (Fig.S1). To shed light on this phenomenon the genome sequences and the phenotypes of the two strains were compared. A third R20291 strain used by Novartis (CRG3661) was included for comparative purposes.

Genomic DNA from all three strains was subjected to Illumina paired-end sequencing and the reads assembled and aligned with the reference genome sequence (Accession number: FN545816). This analysis identified six single nucleotide polymorphisms (SNPs) across all three strains that deviate from the reference sequence, alongside thirteen insertions and eleven deletions (Table 1). In addition to the mutations that were conserved across all three strains, CRG0825 possessed three deletions and two SNPs that were not present in the reference or CRG2021 sequence, whilst the CRG3661 possessed three unique SNPs (Table 1). CRG2091 did not possess any unique mutations compared with the reference genome sequence.

**Table 1:** Genomic mutations of the three R20291 stocks compared with the reference genome sequence.

Position	Gene	Locus Tag	Type	Reference ID	Mutated ID	AA substitution
[1] Common to all three strains						
132924	*met-tRNA*		Insertion	–	A	
132939	*met-tRNA*		SNP	G	T	
132955	*met-tRNA*		SNP	C	A	
132958–59	*met-tRNA*		SNP	TT	CG	
143463	Intergenic		Insertion	–	A	
206399	Intergenic		Insertion	–	A	
581481	Intergenic		Insertion	–	A	
581488	Intergenic		Insertion	–	A	
581495	Intergenic		Insertion	–	A	
1564432	Intergenic		Deletion	A	–	
1568676	Ruberythrin	CDR20291_1323	SNP	C	A	Gln138Lys
1578167	Intergenic		Deletion	T	–	
1578203	Intergenic		Insertion	–	A	
1592813	Intergenic		SNP	A	T	
1864417	Pseudogene	CDR20291_1576	Insertion	–	T	Ser7Frameshift∗
1899596	Intergenic		Deletion	A	–	
2235738	Membrane protein	CDR20291_1913	Deletion	T	–	Val83Frameshift
2262060	Intergenic		Insertion	–	A	
2264190	Intergenic		Deletion	T	–	
2298111	Intergenic		Insertion	–	T	
2361948	Intergenic		SNP	C	A	
2361957	Intergenic		Insertion	–	A	
2367942	Intergenic		Insertion	–	T	
2578157	Intergenic		Deletion	T	–	
2674744	Intergenic		Deletion	T	–	
2680787	Intergenic		Insertion	–	T	
2772179	Pseudogene∗∗	CDR20291_2368	Deletion	T	–	
3077986	Intergenic		Deletion	A	–	
3162098	Intergenic		Deletion	T	–	
3361915	Intergenic		Deletion	A	–	
[2] Specific to CRG0825						
9694	*rsbW*	CDR20291_3551	SNP	G	T	Gly82Val
358260	*rbsK*	CDR20291_0302	Deletion	A	–	Met57Stop
2077305	Intergenic	(CDR20291_1777 to CDR20291_1778)	Deletion	C	–	
2120669	*vncR*	CDR20291_1806	SNP	A	G	Asp202Gly
2881467	TCS-HK∗∗∗	CDR20291_2456	Deletion	T	–	Leu434Stop
[3] Specific to CRG3661						
1340128	*codY*	CDR20291_1115	SNP	T	A	Try146Asn
3292465	*gntR* regulator	CDR20291_2781	SNP	T	C	Ile58Met
3472928	Intergenic	(CDR20291_2929 to CDR20291_2928)	SNP	G	A	

The region encompassing the mutation was aligned with multiple *C. difficile* genome sequences using NCBI Blastn. ∗Insertion here results in a frameshift mutation to a full-length pseudogene encoding an 87 AA protein. This gene without mutation encodes only 6 AAs. ∗∗Putative competence membrane protein ∗∗∗TCS-HC: Two-component system histidine kinase. The gene resides immediately downstream of an adjacent gene (CDR20291_2457) encoding a putative response regulator.

**Fig. 1:**
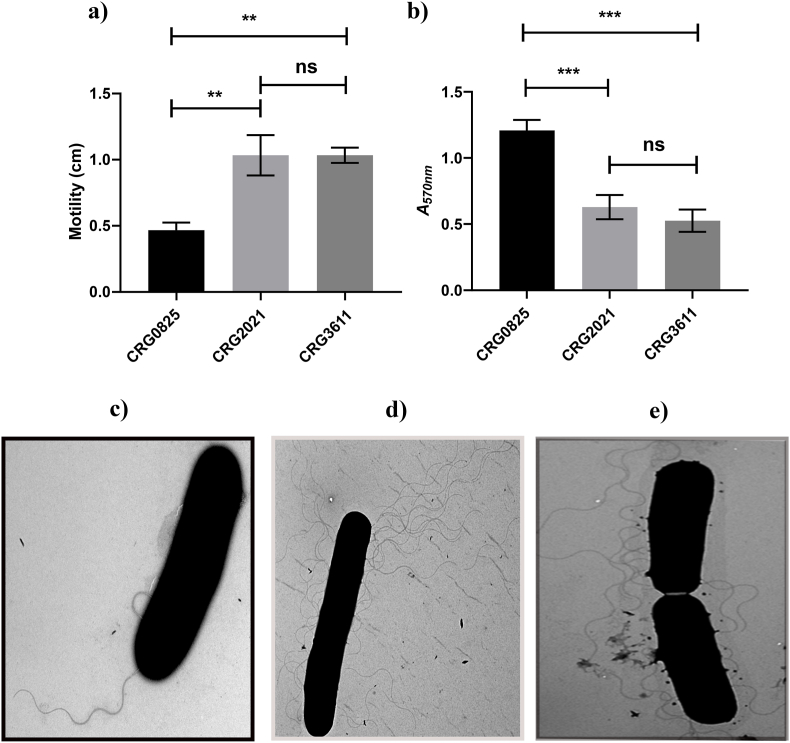
Motility, biofilm formation and Transmission electron microscopy of R20291 derivatives. a) The three derivatives of *C. difficile* R20291 were assessed for their motility characteristics using a swarming motility assay. Motility is represented by the distance travelled from the initial inoculum to the outermost edge of the ensuing halo following 48 h incubation. **b**) The three R20291 derivatives were assessed for their propensity to form biofilms by means of a biofilm assay. Biofilm production is represented by the enumeration of crystal violet dye extracted from 120 h broth cultures. Data represent the mean ± SD of three independent experiments. Statistical significance according to One-way ANOVA followed by the Dunnet's multiple comparison test. P = ∗∗<0.01; P-∗∗∗<0.001. Transmission electron microscopy analysis of **c)** CRG0825; **d)** CRG2091; **e)** CRG3661.

Flagella likely play an important role in the conjugation process. We had previously noted that CRG0825 carried a single, polar flagella [7]. A separate study suggested that CRG2091 was peritrichously flagellated [8]. These differences were confirmed here using Transmission Electron Microscopy (TEM) and extended to establish that CRG3661 was also peritrichously flagellated (Fig. 1c–e). Further analysis demonstrated that CRG0825 exhibited an approximate 50% reduction in swimming motility relative to the other two strains (Fig. 1a). Moreover, consistent with its reduced motility, strain CRG0825 was also found to show a greater propensity to form biofilm, as measured by a biomass formation using crystal violet [9], than strains CRG2021 and CRG3661 (Fig. 1b).

**Fig. 2:**
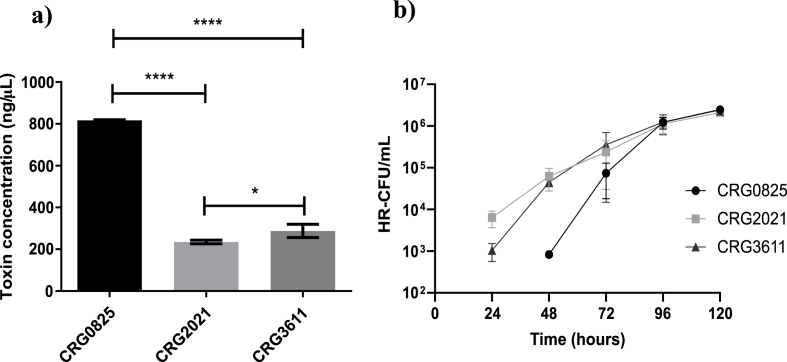
Toxin and sporulation profiles of R20291 derivatives. The three derivatives of R20291 were assessed for **a)** Their ability to produce and secrete toxin through a combined ELISA for TcdA and TcdB on sterile-filtered 72 h supernatants **b)** Their ability to form heat-resistant endospores (heat-resistant colony forming units HR-CFU/mL) across six time-points between 0 and 120 h. Data represent the mean ± SD of three independent experiments. Statistical significance according to One-way ANOVA followed by the Dunnet's multiple comparison test (P = ∗<0.05; ∗∗∗∗<0.0001).

Other studies have linked flagella-mediated motility with toxigenesis in *C. difficile* [10]. Therein, inactivation of early-stage flagella genes led to increased toxin production corresponding with enhanced *in vivo* virulence, whilst inactivation of late-stage flagella genes had the opposite effect [11,12]. Accordingly, we assessed the levels of toxin production in the three strains using a commercial ELISA kit on 72 h filter-sterilised supernatants as described previously [13]. An approximately 3.5-fold increase in toxin production was observed for the CRG0825 compared with the CRG2021 strain which produced around 22% less combined Toxin A/B than CRG3661 (Fig. 2a).

Having established that genetic differences between the strains had affected the important virulence determinants of motility and toxin production, we tested to see whether the capacity to form endospores had been altered as spores represent a critical attribute of disease transmission. Under the conditions tested it was established that the final titre of spores obtained from 96 h onwards was the same regardless of the strain (Fig. 2b). The first appearance of spores in cultures of CRG0825, however, was significantly delayed by some 24 h compared to the other two strains (Fig. 2b).

Finally, the growth performance of each strain was compared. On complex medium, CRG0825 grew to a lower optical density (OD) during the exponential and stationary growth phases than the CRG3661 or CRG2021 strains, where the greatest disparity was observed between the CRG0825 and CRG3661 (Fig S2a). Intriguingly, the observed difference was reversed when the strains were cultured on minimal medium containing either glucose, fructose or mannitol (1% w/v) as the primary carbohydrate source (Fig. S2b-d).

The net result of our analysis was that the two R2091 strain CRG0825 and CRG2021 exhibit significant phenotypic differences. Aside from its greater efficiency as a recipient in conjugations with *E. coli* donor strains, CRG0825 was less motile and exhibited a greater propensity to form biofilm, as measured by a standard crystal violet assay. These differences may represent a consequence of its apparent possession of a single, polar flagella as opposed to the peritrichous flagella of CRG2021, as visualised under TEM. CRG0825 was also shown to produce higher levels of toxins, delayed sporulation and different growth characteristics to CRG2021 on rich and minimal media. The cause of these phenotypic differences are undoubtedly the SNPs and Indels present in CRG0825. A number of pivotal questions emerge from these observations.

**What are the specific causes of the observed changes in phenotype?** Many of the changes appear linked to flagella and motility, yet none of the five mutations in CRG0825 reside directly within, or flank any known flagella genes, and are most likely impinging on the regulation of these processes. Moreover, regulation of flagella, toxin production and virulence are known to be linked in *C. difficile* [[10], [11], [12]]. Three of the four non-synonymous SNPs present in CRG0825 do indeed affect apparent regulatory genes, namely *vncR,* TCS HK and the anti-sigma factor *rsbW.* Two of the three non-synonymous changes in CRG3661 are also in regulatory genes, *codY* and a *gntR* family regulator. However, to pin down exactly which SNP(s) or Indels, are responsible for the observed phenotypic differences between CRG0825 and CRG2021, for instance, would require a considerable effort in which all combinations of mutation would need to be generated in allele replacement experiments during which the generation of additional changes would need to be excluded.

**How did these changes arise?** Following their discovery, correspondence with Val Hall at the ARU revealed that at the time R20291 was sent to Nottingham, it was routine ARU practice to “*keep a small number of isolates that are used as internal lab controls on agar plates, sub-culturing weekly plate-to-plate and retrieving fresh cultures from the original vial periodically”.* The sequence presented here is from Nottingham's −80 °C, Master seed bank (red tube) prepared immediately on receipt of the strain in 2006. We can conclude, that during the repeated subculture of the R20291 stock at the ARU in 2006, the 5 described mutations arose. This practice of maintaining a stock plate no longer takes place at the ARU. The consequences of subculturing have previously been noted in the case of the *C. difficile* strain 630, where deliberate, repeated subculture led to the emergence of two very different cell lines (630Δerm and 630 E) carrying distinct SNPs, inversions and deletions and which exhibited differences in motility, spore formation and toxin production, as well as overall virulence in the hamster model of infection [14].

**What are the lessons to be learnt?** The take home message of this investigation is that stock cultures need to be appropriately maintained. At Nottingham, a traffic light system is used to store bacterial cultures. Upon receipt of cultures, aliquots of cells (never single colonies) are used to inoculate an overnight which following addition of 10% glycerol is allocated to three 2 ml screwed capped tubes with a red, amber and green coloured cap insert and stored at −80 °C. Red tubes remain untouched and are stored in a separate freezer. Green tubes represent the working stock which may be restocked from the amber tube where necessary.

The R20291 strain maintained at LSHTM has a genome sequence consistent with the sequence held at GenBank (Accession number: FN545816). The differences listed in Table 1 are common to all strains, and therefore likely represent errors in the original sequence. The strain CRG3661 began its journey at LSHTM and found its way to Novartis via the Trevor Lawley laboratory at the Sanger Institute, and thence to Nottingham. It is not clear when its three mutations arose. The Nottingham CRG0825 apparently arose as a result of repeated subculture at the ARU.

**What is the way forward?** It is clearly advisable that the genome sequences of any stock culture of any bacterial strain stored in a laboratory should be confirmed, regardless of source, prior to use. This principal should equally apply to any mutant derivative of a strain made by whatever means, to ensure that additional SNPs/Indels have not arisen.

On the specific subject of studies dealing with R20291, it is important that experimentalists are aware of the differences between the strain lineages discussed here, and that the strain used is made clear in any meeting presentation or published article. CRG0825 is a widely distributed strain, owing to its superior conjugative efficiencies and its usage in the development of AE mutagenesis technologies [6]. The absence of polymorphisms specific to CRG2021, however, suggests that this strain is the closest ancestor of the original R20291 clinical isolate. Although the lack of a characterised uracil auxotroph, in addition to difficulties concerning conjugal transfer, formally reduced its attractiveness compared to CRG0825, recent advances that improve gene transfer frequencies [15,16] and the advent of multiple CRISPR-Cas methodologies for use in *C. difficile* research [[17], [18], [19], [20]], have improved the tractability of CRG2021 to genetic studies. For those researchers who wish to use AE technologies based on *pyrE* [6], the requisite auxotrophic uracil mutant of CRG2021 is available from www.plasmidvectors.com.

## Author contributions

Conceived the experiments: NPM. Performed the experiments: JM, TWB and PI. Undertook genome sequence determination and analysis: SP, TWB and NPM. Analysed the data: JM, TWB, PI, SAK and NPM. Wrote the paper: TWB and NPM. All authors read and commented on the final manuscript.

## Declaration of competing interest

The authors declare no conflicts of interest.
