# Supplementary material for: What's a SNP between friends: The lineage of Clostridioides difficile R20291 can effect research outcomes
Source: Anaerobe. 2021 Oct;71:102422. doi: 10.1016/j.anaerobe.2021.102422 (PMC8556159; doi:10.1016/j.anaerobe.2021.102422)
Supplement: Multimedia component 1 [file mmc1.docx]

**What’s a SNP between friends: the lineage of *Clostridioides difficile* R20291 can effect research outcomes.**

Jorge Monteford^1^*, Terry W. Bilverstone^1,2^*, Patrick Ingle^1,2^, Sheryl Philip^1,3^, Sarah A. Kuehne^1,4^, Nigel P. Minton^1,2^.

* These authors contributed equally.

^1^ Clostridia Research Group, BBSRC/EPSRC Synthetic Biology Research Centre (SBRC), School of Life Sciences, Centre for Biomolecular Sciences, The University of Nottingham, Nottingham, NG7 2RD, UK.

^2^ NIHR Nottingham Biomedical Research Centre, Nottingham University Hospitals NHS Trust and the University of Nottingham, Nottingham, NG7 2RD, UK.

Corresponding author: nigel.minton@nottingham.ac.uk

*Current Address:*

^3^Biocatalysts Limited, Unit 1, Cefn Coed, Parc Nantgarw, Cardiff, CF15 7QQ, UK,

^4^Oral Microbiology Group, School of Dentistry and Institute of Microbiology and Infection, College of Medical and Dental Sciences, The University of Birmingham, Birmingham, B5 7EG, UK.

**Supplementary material**

**Table S1: *C. difficile* strains used in this study**

| **Strain** | **Description** |
| --- | --- |
|  |  |
| CRG0825  CRG2021 | R20291 sent to Nottingham by Val Hall in 2006 from the Anaerobe Reference Unit (ARU), Cardiff, UK.  R20291 sent to Nottingham by Lisa Dawson in 2010 from the laboratory of Brendan Wren at the London School of Hygiene and Tropical Medicine (LSHTM), UK. Originally obtained from the ARU, Cardiff, UK. |
|  |  |
| CRG3661 | R20291 sent to Nottingham by Meera Unnikrishnan in 2013 from Novartis, Sienna, Italy. Sent to Novartis by Trevor Lawley at the Sanger Institute, Cambridge, UK. Originally obtained from the Brendan Wren laboratory at LSHTM, London UK. |
| CRG1375 | R20291 (CRG0825) *spo0A* ClosTron mutant [1]. |
|  |  |

**Experimental**

**Bacterial Strains and Growth Conditions**

Strains were routinely cultured anaerobically at 37 °C in an anaerobic MACS1000 workstation (Don Whitely, Yorkshire, UK) in BHIS (Brain Heart Infusion supplemented with yeast extract [5 mg.ml−1] and L-cysteine [0.1% w/v]) medium supplemented with d-cycloserine (250 μg.ml−1), cefoxitin (8 μg.ml−1) and thiamphenicol (15 μg.ml−1) or Em (10 μg.ml−1) where appropriate.

**Comparative conjugations**

Conjugations of shuttle vectors into *C. difficile* R20291 were performed as described in [2]. Briefly, *E. coli* CA434 donor strains harbouring either pMTL82151, pMTL83151 or pMTL84151 were grown overnight in LB supplemented with chloramphenicol and kanamycin. From which, aliquots (1ml) were pelleted, washed in PBS and resuspended in 200 µl of *C. difficile* R20291 cultures grown anaerobically overnight in BHIS broth. The resulting conjugal mixtures were spotted onto BHIS plates lacking antibiotics and incubated anaerobically for 24h. Subsequent growth was resuspended in PBS (500µl) and spread onto BHIS plates supplemented with d-cycloserine and cefoxitin, both with and without thiamphenicol. After 72h, Thiamphenicol (Tm) resistant (^R^) CFU.ml^-1^ and total *C. difficile* CFU.ml^-1^ values were determined from the subsequent growth of *C. difficile* R20291 strains in the presence (Tm^R^) or absence (total) of thiamphenicol. Conjugation efficiency was calculated as the Tm^R^ CFU.ml^-1^ divided by total *C. difficile* CFU.ml^-1^.

**24h growth curve**

The growth characteristics of *C. difficile* R02921 was assessed by manual growth curve. Therein, colonies of *C. difficile* R20291 were subcultured into fresh BHIS broth in an Anaerobic Work Station (Don Whitley, UK), at 37°C with an anaerobic atmosphere comprising 80% N_2_, 10% H_2_ and 10% CO_2_. The resultant cultures were then diluted 1/100 in fresh BHIS and grown to an optical density value (OD_600nm)_ of 0.2-0.5. This generated replicates of each strain with similar starting OD values for downstream growth assessment. Once target OD values had been reached, the cultures were diluted 1/100 in fresh medium incubated for 24h. 1ml of sample was taken for each replicate at hourly intervals which was then assessed for its optical density using a Novaspec II spectrophotometer (Geminibv, Netherlands).

**Motility assay**

The motility of *C. difficile* R20291 derivatives was assessed by swimming motility assays as previously described [3]. Therein, single colonies of R20291 were isolated using a toothpick and stabbed onto the centre of semi-solid BHIS plate containing 0.3% (w/v) agar. Following 48h incubation as described above, the diameter of the ensuing halo was measured. Motility is represented as the distance between the centremost and outermost points of detected colonisation (cm).

**Crystal Violet, Biofilm Assay**

The assay was undertaken essentially as described by Dapa and co-workers [4]. A starter culture of *C. difficile* was prepared by inoculating fresh BHIs broth containing 0.1M glucose with an overnight culture of the desired strain in a 1:100 dilution. A 1ml aliquot of this culture added to each well of a 24-well Tissue culture plate (Costar,USA) and incubated anaerobically for 120h. Plates were pre-reduced in the anaerobic cabinet for 48h prior to use. To avoid liquid evaporation, each plate was wrapped in parafilm. Following incubation, wells were washed with PBS and the plate allowed to dry for 10m. The biofilm was stained with 1ml of filter-sterilised 0.2% (w/v) crystal violet solution per well and incubated for 30m at 37°C under anaerobic conditions. The staining solution was removed and the wells were washed twice with PBS. The plate was removed from the anaerobic cabinet and 1ml methanol was added to the wells for the removal of the dye from the biofilm and it was incubated for 30m at room temperature. The methanol extracted dye was diluted 1:1, 1:10 and 1:100 and the absorbance A570 was measured with Ultrospec 500 pro spectrophotometer.

**Sporulation assay**

Cultures were generated for each strain with similar staring OD values as described for the 24h growth curve. For the sporulation assay, cultures were incubated for a 120h period. Samples were taken at 24h intervals which were heated at 65°C for 30m in order to eradicate vegetative cells and diluted 1x10^-1^-1x10^-8^ before plating onto BHIS supplemented with 0.1% taurocholate germinant. Spores were then enumerated for each R20291 derivative alongside a ClosTron insertional mutant for the master regulator of sporulation *spo0A* [1].

**Detection of combined TcdA and TcdB**

Combined TcdA and TcdB was detected as previously described [5]. Cultures of each strain were collected after 72h, the OD measured, and normalised to the lowest OD value. Normalised samples were centrifuged and the supernatant passed through a 0.22µm filter and frozen at -20°C until required (<1 week). Thawed samples were diluted 1X10^1^ – 1X10^8^ in sterile PBS and processed for ELISA quantification of total TcdA and TcdB using a C. DIFFICILE Tox A/B II detections kit (TechLab, USA) according to the manufacturer’s instructions. Absorbance values were converted into toxin concentration by determining the R2 value of the assay’s standard curve by running a range of defined combined TcdA and TcdB toxin standards from 0-125ng/ml (The Native Antigen Company).

**Genome Sequencing**

Chromosomal DNA of each strain was prepared and subjected to Illumina paired-end sequencing by DeepSeq (University of Nottingham) using the MiSeq v3 600 platform. Paired reads were trimmed, before mapping the trimmed reads to the reference genome sequence for R20291 (Accession number: FN545816) using the quality-based variant detection workflow from CLC Genomics Workbench (Qiagen, Germantown, USA). The software was then used to identify single nucleotide variations (SNVs), insertions and deletions compared with the reference genome sequence. Sequencing reads were deposited to the NCBI Sequencing Reads Archive with the Bioproject accession PRJNA689976 and the following individual accession numbers: CRG2021 (SRR13366486); CRG0825 (SRR13366485); CRG03661 (SRR13366484).

**Supplementary Figures**


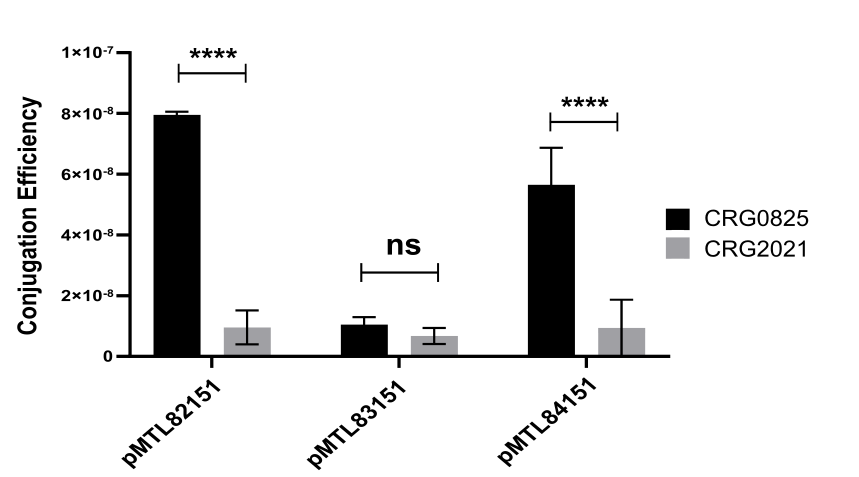


**Fig S1:** **Comparative conjugation efficiencies of plasmid transfer from *E. coli* CA434 into *C. difficile* R20291 stocks CRG0825 and CRG2021.** Conjugations from *E. coli* CA434 strains harbouring the indicated shuttle vectors, differing only in the Gram-positive replicon present, into *C. difficile* R20291 CRG2021 (grey bars) and CRG0825 (black bars) were performed as indicated in Materials and Methods. Conjugation efficiency was calculated as thiamphenicol resistant CFU.ml^-1^ divided by the total recipient *C. difficile* R20291 CFU.ml^-1^. Data represent the mean ±SD of three independent experiments. Statistical significance was determined using multiple unpaired t-tests. P=****<0.0001; ns= not significant.


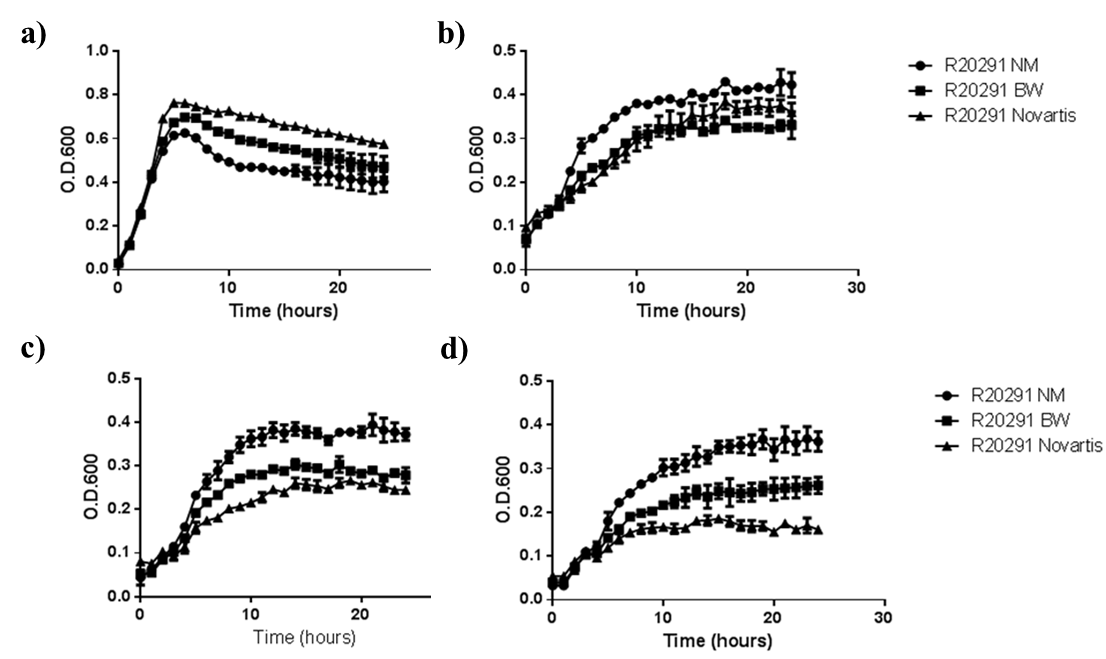


**Figure S2: Comparison of growth characteristics between the three derivatives of R20291.** R02921 strains were grown for 24h in a) BHIS broth; b) CDMM 1% (w/v) glucose; c) CDMM 1% fructose; d) CDMM 1% mannitol. Data points indicate the mean ±SD of three independent experiments.

**References**

1. Heap J.T., Kuehne S.A., Ehsaan M., Cartman S.T., Cooksley C.M., Scott J.C., Minton N.P. *J Microbiol Methods*. The ClosTron: Mutagenesis in *Clostridium* refined and streamlined. 2010;**80(1):**49-55.
2. Woods C., Humphreys C.M., Rodrigues R.M., Ingle P., Rowe P., Henstra A.M., Köpke M., Simpson S.D., Winzer K., Minton N.P. A novel conjugal donor strain for improved DNA transfer into *Clostridium* spp. *Anaerobe*. 2019;**59:**184-191.
3. Baban S.T., Kuehne S.A., Barketi-Klai A., Cartman S.T., Kelly M.L., Hardie K.R., Kansau I., Collignon A., Minton N.P. The role of flagella in *Clostridium difficile* pathogenesis: comparison between a non-epidemic and an epidemic strain. *PLoS One*. 2013;**8(9):**e73026.

4. Ðapa T., Leuzzi R., Ng Y.K., Baban S.T., Adamo R., Kuehne S.A., Scarselli M., Minton N.P., Serruto D., Unnikrishnan M. Multiple factors modulate biofilm formation by the anaerobic pathogen *Clostridium difficile*. *J Bacteriol*. 2013;**195(3):**545-55.

5. Bilverstone T.W., Kinsmore N.L., Minton N.P., Kuehne S.A. Development of *Clostridium difficile* R20291ΔPaLoc model strains and in vitro methodologies reveals CdtR is required for the production of CDT to cytotoxic levels. *Anaerobe.* 2017;**44:**51-54.
